# High-strength cellulose nanofiber/graphene oxide hybrid filament made by continuous processing and its humidity monitoring

**DOI:** 10.1038/s41598-021-93209-5

**Published:** 2021-06-30

**Authors:** Hyun Chan Kim, Pooja S. Panicker, Debora Kim, Samia Adil, Jaehwan Kim

**Affiliations:** Creative Research Center for Nanocellulose Future Composites, Inha University, 100 Inha-ro, Michuhol-ku, Incheon, 22212 Republic of Korea

**Keywords:** Mechanical engineering, Organic-inorganic nanostructures, Composites

## Abstract

Human-made natural-fiber-based filaments are attractive for natural fiber-reinforced polymer (NFRP) composites. However, the composites' moisture distribution is critical, and humidity monitoring in the NFRP composites is essential to secure stability and keep their life span. In this research, high strength and humidity sensing filament was developed by blending cellulose nanofiber (CNF) and graphene oxide (GO), wet-spinning, coagulating, and drying, which can overcome the heterogeneous mechanical properties between embedded-type humidity sensors and NFRP composites. The stabilized synthesis process of the CNF-GO hybrid filament demonstrated the maximum Young's modulus of 23.9 GPa and the maximum tensile strength of 439.4 MPa. Furthermore, the achieved properties were successfully transferred to a continuous fabrication process with an additional stretching process. Furthermore, its humidity sensing behavior is shown by resistivity changes in various temperature and humidity levels. Therefore, this hybrid filament has excellent potential for in-situ humidity monitoring by embedding in smart wearable devices, natural fiber-reinforced polymer composites, and environmental sensing devices.

## Introduction

Recently, human-made natural-fiber-based filaments have gained attention because natural fibers are short, and when they are made a yarn, their mechanical properties are degraded^1–4^. Human-made natural-fiber-based filaments are attractive for wearable devices and natural fiber-reinforced polymer (NFRP) composites owing to their environment-friendly and renewable behaviors. Cellulose is an abundant natural polymer that constitutes structures of higher plant cells^5^. The hierarchical structure of cellulose leads to its high mechanical properties with flexibility. Nanocellulose, a cellulose nanoparticle with at least one dimension less than 100 nm, has been utilized for human-made natural-fiber-based filaments using wet-spinning, dry-spinning, melt-spinning, and extrusion^1–4^. Recently, a cellulose nanofiber (CNF)-based filament exhibited its Young's modulus of 37 GPa and tensile strength of 540 MPa^6–8^.

Although they show outstanding mechanical properties, the filament is sensitive to moisture due to the CNF's hydrophilicity. Thus, humidity detection capability is suitable for NFRP composites and wearable devices such that the composites or devices can adaptively adjust their behaviors. Furthermore, in the NFRP composites area, moisture distribution through the structure is a critical issue. The moisture could induce the micro-cracks and weakens the matrix surface interface, leading to the failure of composites^9^. Therefore, humidity monitoring for the NFRP composites is essential to secure stability and keep their life span. With this background, embedded-type humidity monitoring sensors have been studied to avoid the composites' unexpected failure. Microelectromechanical systems with water-sensitive functional polymer were proposed to place into structures^10^. Embedded fiber Bragg grating sensors to detect water contamination in the composite elements also were reported^11^. However, the sensors can cause defects or fatigue failure in composite materials due to heterogeneous mechanical properties. Therefore, the ideal embedded sensors demand high sensitivity and appropriate mechanical properties matched with the target composites.

On the other hand, graphene oxide (GO) is taken for the proposed sensing functionality. Since GO has good electrical properties and surprising mechanical properties, it has drawn attention in many fields, such as sensor, biomedicine, and nanotechnology^12–15^. GO has a large surface area containing many hydrophilic functional groups, such as hydroxyl, carboxyl, epoxy bridge, and carbonyl. The presence of these functional groups makes GO readily dispersible in water. Thus, it provides convenient access to fabricate nanocomposites with other hydrophilic polymer materials by solution mixing. Since GO is extracted from a cheap source, graphite has an exceptional price benefit over CNTs. Thus, it is natural to invent GO/CNF composites since they are renewable and multifunctional. According to previous studies for films, GO has been used to reinforce sodium carboxymethyl cellulose matrices, add sensor functionality with cellulose nanocrystals, and design flexible energy applications with regenerative cellulose^14,15^. Researches for filament formation have also been conducted: cellulose, GO, and barium ions were blended and achieved 580 MPa tensile strength; nanofibrillated cellulose and GO were hybridized and showed 31.6 GPa Young's modulus and 416.6 MPa tensile strength^16,17^. However, since their fabrication process was manually operated non-continuous that took over 48 h, it is not suitable for mass production. A recent report for continuous filament fabrication by blending cellulose nanocrystals and GO showed relatively low tensile strength and strain-at-break of 87.1 MPa and 3%^18^. Thus, achieving robust mechanical properties through continuous processes remains a challenge.

A total solution for fabricating high strength and humidity sensing CNF-GO (CNGO) hybrid filament is proposed by optimizing the mixing ratio between CNF and GO and adopting a continuous fabrication process. The hybridization of CNF and GO was conducted by a simple solution mixing method. After successful mixing, the CNGO hybrid suspension was wet-spun in a coagulation bath manually. Then, the continuous fabrication process was performed in the same way by adding a stretching step. The prepared CNGO hybrid filaments were studied in terms of morphology, surface chemistry, thermal characteristic, mechanical properties, and humidity sensing performance.

## Experimental procedure

### Materials

According to the previous report, the CNF suspension was prepared, combining a 2,2,6,6-tetramethylpiperidine-1-oxylradical (TEMPO) oxidation and aqueous counter collision (ACC) method with centrifugal fractionation^19^. The ACC method uses encountered water jets with a collision pressure of 200 MPa to selectively break up only weak hydrogen bonds without destroying solid bonds in the intermolecular structure of the cellulose chain. Thus, ACC is a strategic and efficient physical method that can isolate long CNF from pretreated cellulose resources^20^. Based on the optimized results of the previous study, the preparation of CNF includes 60 min of TEMPO treatment time, 30 passes of ACC process, and centrifugal fractionation at 45 k rpm for 4 h. The hardwood pulp (bleached acacia kraft pulp) was purchased from Hansol paper, South Korea, and the CNFs used in the study have average width and length of 2.0 ± 0.6 nm and 639 ± 387.3 nm, respectively^19^. An aqueous dispersion of single-layer graphene oxide was purchased from Graphene Supermarket, USA. The graphene oxide flake size ranges from 0.3 to 0.7 μm with a 20% of oxygen composition. CaCl_2_ and Citric acid (99.9%) were purchased for coagulants from Samchun Chemical and Sigma-Aldrich.

### Preparation of CNGO hybrid suspension

First of all, CNGO hybrid suspension was prepared by mixing the CNF and GO diluted to 0.5 wt% in DI water separately and mechanically stirred for 2 h by controlling the mixture ratio between CNF and GO suspension. Then, the mixed CNGO hybrid suspension was densified from 0.5 to 2 wt% through slow water evaporation under 40 °C with the mechanical stirring of 500 rpm for 48 h. The mixture ratio represents the GO weight percent to the total weight of CNGO hybrid filaments: 1, 3, 5, and 7 wt%, annotated as CNGO 1, CNGO 3, CNGO 5, CNGO 7.

In the previous study, an optimal CNF concentration for wet spinning was founded to be approximately 2 wt%, based on Fick's law^21^. The high concentration suspension impedes the ion exchange effect in the wet spinning process and induces the self coagulation due to intermolecular forces between nanomaterials such as van der Waals forces and electrostatic interaction^22^. On the other hand, a low concentration of suspension assigns a reason for the unstable structure after wet spinning. Thus, the concentration of CNGO hybrid suspension was designed to 2 wt% via slow water evaporation.

### CNGO hybrid filaments preparation

**Figure 1:**
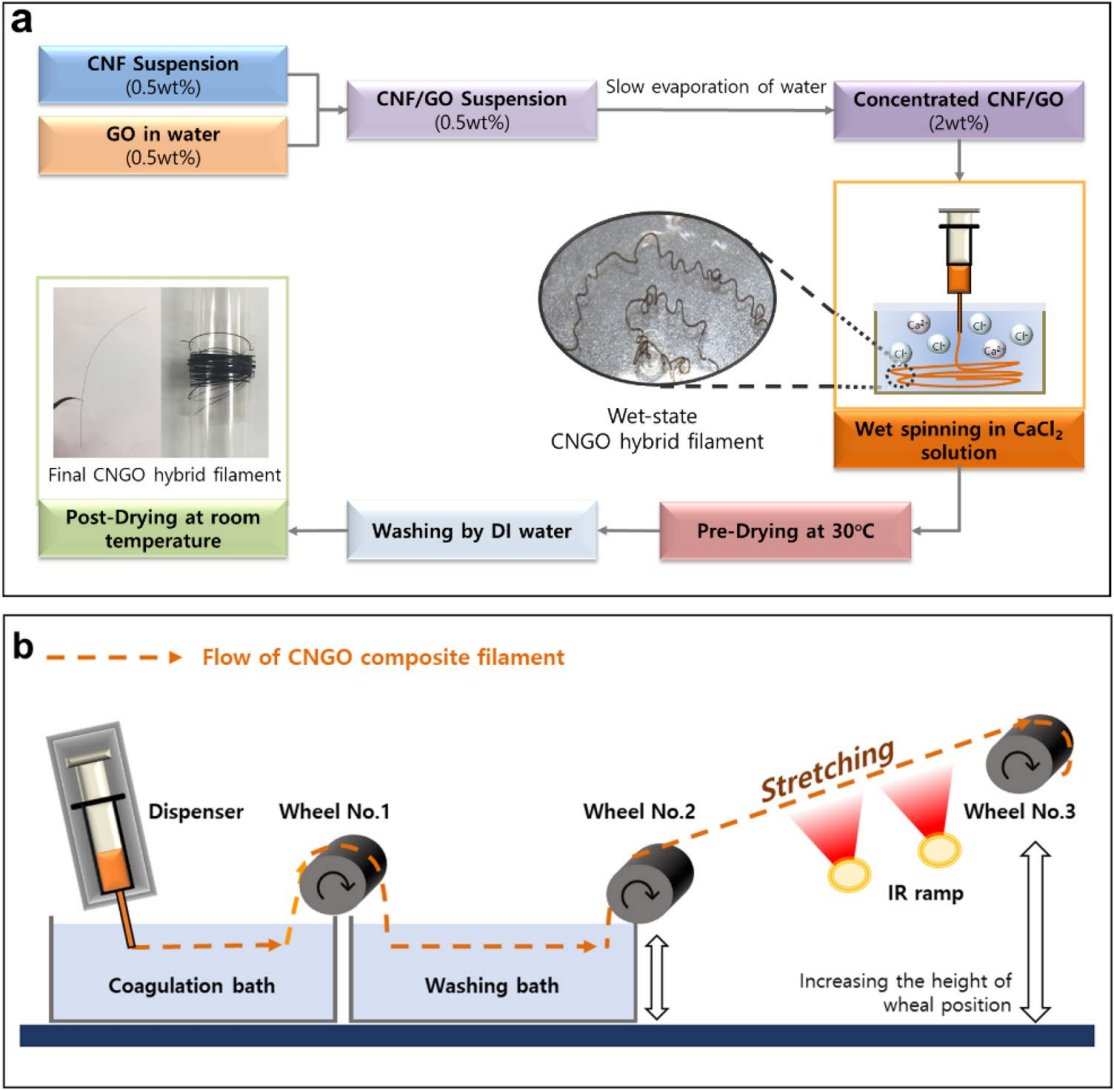
Schematic diagrams of the CNGO hybrid filament preparation: (**a**) the manual process and (**b**) the continuous process.

The CNGO hybrid filaments were prepared in two methods: manual process and continuous process. The manual process is the initial wet-spinning method, and its results were compared with the continuous process, the designed process in this paper. Figure 1a shows a schematic diagram of the manual process for the CNGO hybrid filament preparation. The process is divided into four steps; coagulation, pre-drying, washing, and post-drying. First, the CNGO hybrid suspension was wet-spun using a precision fluid dispenser (SMP3-C, MUSASHI Engineering, Japan) with wet-spinning parameters: 5.11 mm/s spinning speed and 960 μm diameter of PTFE needle. Then, in the coagulation step, the wet-spun filament was kept in the 5 wt% CaCl_2_ solution for 30 min to induce sufficient ion exchange between sodium ions in CNGO hybrid suspension and calcium ions in coagulation solution. Then, the spun filament was collected and pre-dried in a convection oven at 30 °C for 1 h for the next step. Next, the pre-dried filament was washed with DI water for 24 h to remove unreacted CaCl_2_ on the filament's surface. Finally, the filament was post-dried at room temperature. Inset images in Fig. 1a show the wet-state CNGO hybrid filament, fabricated CNGO hybrid filament, and wound filament.

Figure 1b shows schematic diagrams of the designed continuous process for the CNGO hybrid filament preparation. The same wet-spinning system was used, and three wheels were installed to deliver the filament continuously. The wheel was made with polyethylene, and the diameter is 100 mm. The wheel axis was run by a stepping motor (PGM32-NK244, Motor Bank, South Korea) with a motor driver. A proximity detector (PR08-2DN, Autonics, South Korea) was installed above the axis to measure the rpm, and a pulse meter (MP5Y, Autonics, South Korea) converted the detector signal into the wheel's line speed, displayed on the indicator. The wheel, motor, driver, detector, and pulse meter were installed in a stainless-steel frame that can rigidly hold all parts during the operation. A support jack adjusted the wheel height. Before the last winding wheel, two infrared bulbs were installed to dry the wet-state CNGO hybrid filament. The coagulation and washing baths were made with tempered glass.

For the first step in the continuous fabrication process, the prepared CNGO suspension was wet-spun using the precision fluid dispenser with 5.11 mm s^−1^ spinning speeds through the 250 μm diameter PTFE needle. The coagulation was followed in the 0.5 wt% citric acid solution bath. Next, the first wheel drew the wet-state CNGO filament and moved it to the next washing bath of DI water. The continuous filament movement was maintained via the second wheel by taking it away from the washing bath, and it was stretched between the second and third wheels by differentiating the wheel speeds. At the same time, the stretched filament was thoroughly dried by the infrared lamp during the stretching. Finally, the third wheel winded the stretched and dried filament. The continuous process for the CNGO hybrid filament can be seen in Video 1.

### Characterization

Morphology of the CNGO hybrid filaments was investigated using a scanning electron microscope (SEM, JSM-6400 F, JEOL, JAPAN). SEM specimens were sputtered with a thin platinum layer using ion sputter (K575x, EMITECH, FRANCE).

Fourier transform infrared (FTIR) spectroscopy study was conducted to estimate the interaction between CNF and GO using an FTIR spectrometer (FTS 3000, BIO-RAD lab, USA) ranging from 500 to 4000 cm^−1^. A pallet-type specimen was prepared by grinding the filaments with potassium bromide under pressure.

The surface chemistry was studied by X-ray photoelectron spectroscopy (XPS, K-alpha, Thermo scientific, USA) with an X-ray from monochromatic Al K-alpha radiation. Survey spectra were measured from 0 to 1350 eV in 1 eV steps, and Carbon 1 s spectra were recorded from 280 to 300 eV in 0.1 eV steps with 400 µm of spot size.

Thermal stability was identified by a thermal gravimetric analyzer (TGA, TG209F3, NETZSCH, GERMANY) under a nitrogen environment with a heating rate of 10 °C min^−1^ from 30 to 600 C min^−1^ using samples pristine CNF, GO, and CNGO hybrid.

Tensile tests were carried out to analyze mechanical properties of CNGO 1, 3, 5, and 7 specimens by the standard method, ASTM D-882-97, in 40% RH and 25 ℃ conditions. The tensile speed and initial length were set to 0.005 mm s^−1^ and 10 mm, respectively. The results were averaged from ten samples.

**Figure 2:**
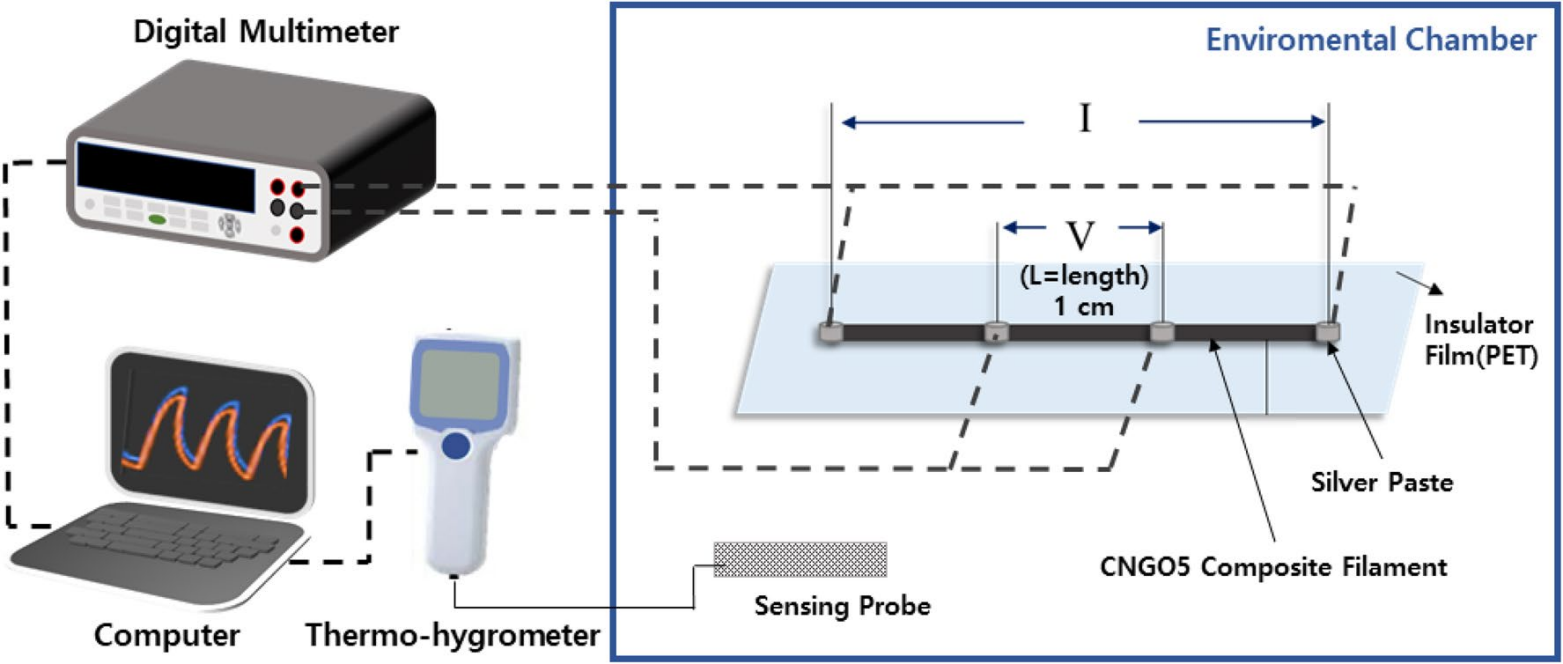
Schematic diagram of the electrical resistance measurement using the four-point probes method.

The four-point probe method was used to measure CNGO hybrid filaments' electrical resistance depending on various temperature and humidity conditions. The resistance measuring technique uses separate two pairs of current-carrying (outer electrode) and voltage-sensing (inside electrodes) to make more accurate measurements than the two-point probes sensing method. Four-point probes are generally used to measure semiconductor materials^23^. Figure 2 shows a schematic diagram of the experimental setup for the four-point probes method. The four electrodes were formed using silver paste with copper wire at intervals of 1 cm on the CNGO hybrid filament. Then, the wires were connected with a digital multimeter (34410A, Agilent, USA). The sample was placed in an environmental chamber (KMN-CTH3-2S, Kwangmyoung Science, South Korea) to measure the resistance under specific temperature and humidity conditions. The following equation can calculate the resistivity,

1 $${\uprho }={\text{R}}\times \frac{A}{L}$$

where ρ is resistivity, R is resistance measured from the four-point proof system, A is the cross-sectional area of CNGO hybrid filament, and L is between two inner probes. In addition, the temperature and humidity were measured by a thermo-hygrometer (TH-05, Daekwang, South Korea). All data from the multimeter and the thermo-hygrometer were collected on the computer at the same time.

## Results and discussion

**Figure 3:**
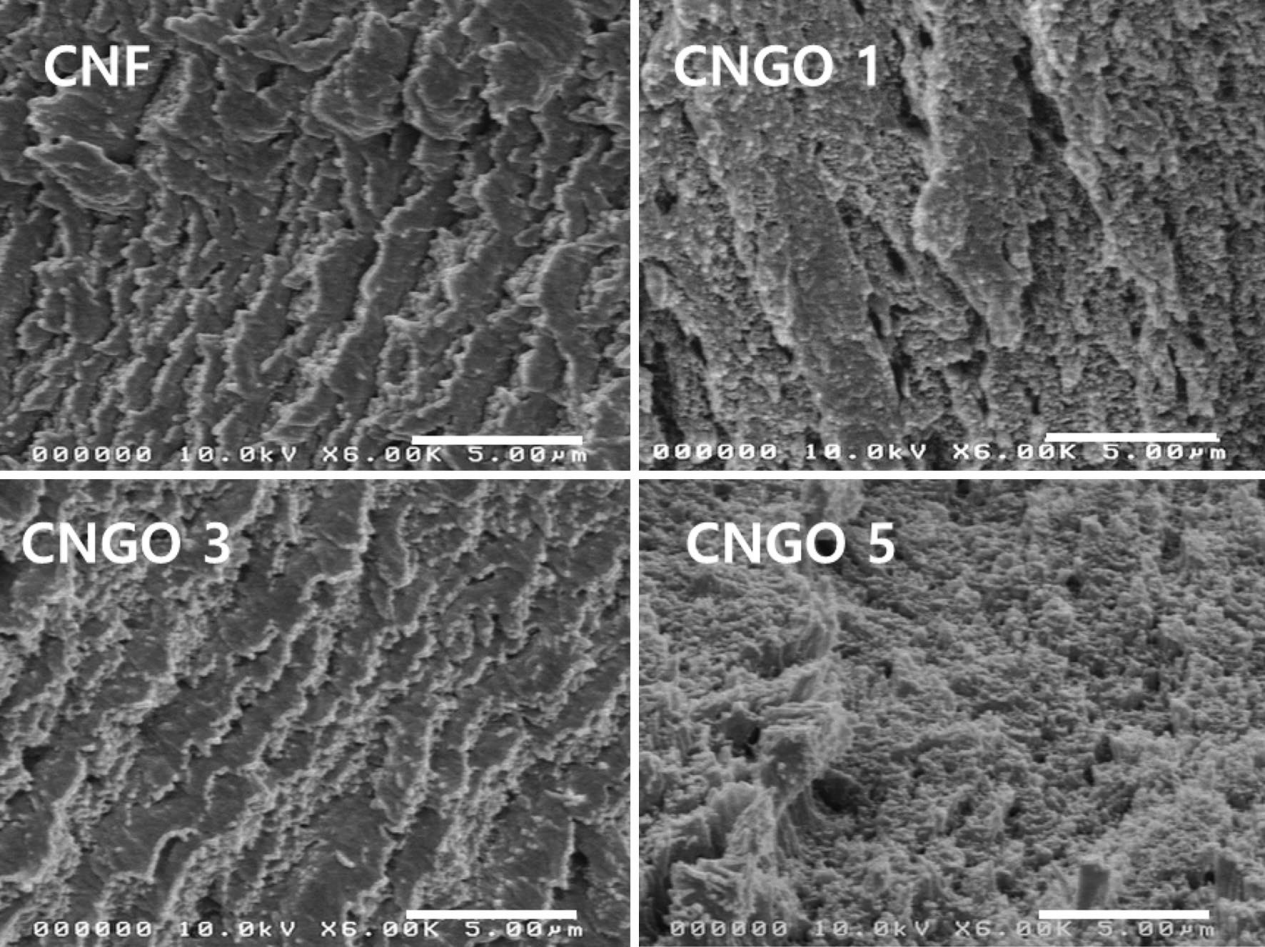
The fractured-surface SEM images of the pristine CNF and CNGO hybrid filament with different GO concentrations.

Figure 3 shows the SEM images of the fracture of the pristine CNF and CNGO hybrid filaments with different GO concentrations. From the images, it can be seen that GO is dispersed uniformly in the composite because its agglomeration was not found. As the GO concentration increases, the fracture surface also becomes smoother in the SEM images. GO has many functional groups such as carboxyl, hydroxyl, carbonyl groups, and epoxy bridges, which enable easy dispersion in the water. CNF also has many hydroxyl groups such that they can be easily mixed, forming well-dispersed CNGO hybrid filament.

**Figure 4:**
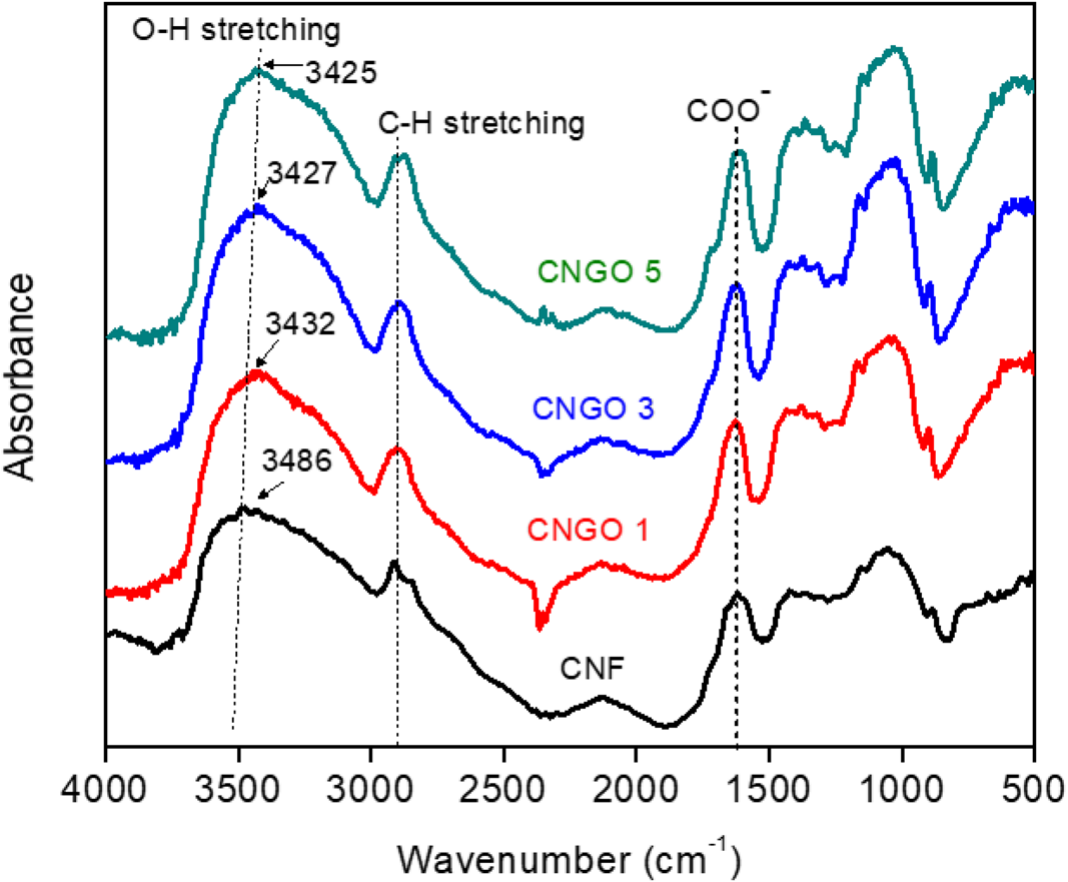
FTIR spectra of the pristine CNF and CNGO hybrid filaments with different GO concentrations.

Figure 4 shows the FTIR results for the pristine CNF and CNGO hybrid filaments with different GO concentrations. All the spectra show similar peaks. The broadband at 3486–3425 cm^−1^ corresponds to O–H stretching vibration, which shifts to a lower wavelength with GO concentration increment due to the intermolecular hydrogen bond between GO and CNF. Peaks at 2900 cm^−1^ indicate the C–H stretching vibration, and 1620 cm^−1^ is assigned to COO^-^ stretching of a carboxyl group. The band around 1365 cm^−1^ is assigned to C–H and C–O bending vibrations. The peak detected at 1054 cm^−1^ arises from the C–O–C pyranose ring antisymmetrical stretching vibration. Identical peaks of CNF and CNGO hybrids filament denote the absence of any noticeable chemical interactions between CNF and GO when mixing^24–26^.

**Figure 5:**
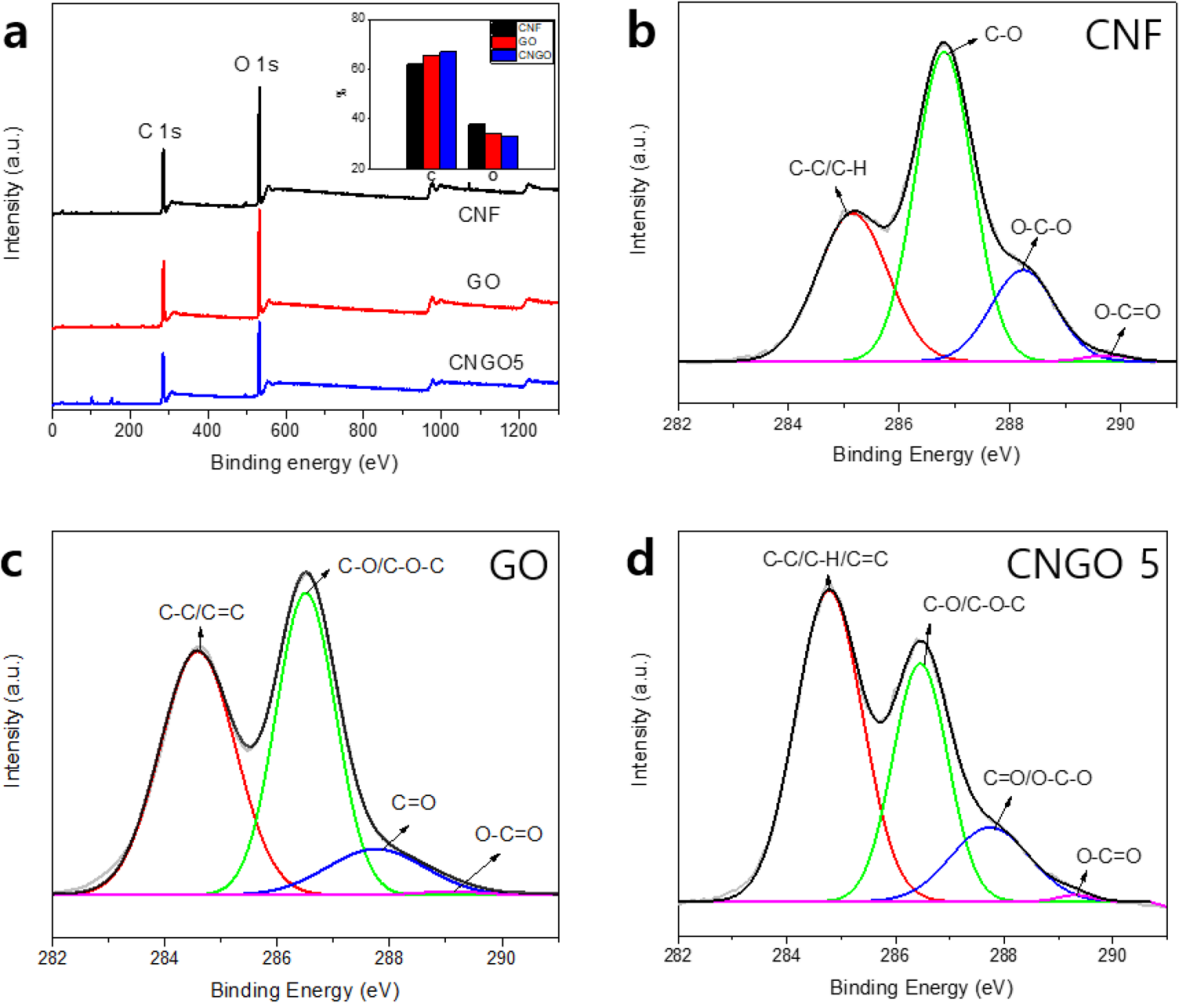
XPS results of the pristine CNF, GO, and CNGO 5: (**a**) XPS survey spectra, (**b**) C 1 s spectra of the pristine CNF, (**c**) C 1 s spectra of GO, and (**d**) C 1 s spectra of CNGO 5.

XPS analysis is a well-known technique for surface characterization. Figure 5a shows XPS survey spectra of the pristine CNF, GO, and CNGO hybrid with similar O 1 s and C 1 s peaks. However, the detailed information is appeared by deconvolution of C1s spectra into several peaks. As shown in Fig. 5b, four different functional groups represent the C 1 s spectrum of the pristine CNF. The first group is C–C/C–H (285 eV), where carbon atoms have no connection with oxygen, and the second group corresponds to C–O (286.8 eV), in which the carbon atom has only one bond with non-carbonyl oxygen. The third group represents O–C–O (288.2 eV), where carbon atoms bond with two non-carbonyl oxygen. The last group is O–C=O (289.5 eV), in which carbon has bonds of carbonyl oxygen and non-carbonyl oxygen^27–29^. Furthermore, the C 1 s spectrum of GO, shown in Fig. 5c, is divided into four groups which are C–C/C=C (284.5 eV) in the aromatic ring, C–O/C–O–C (286.5 eV) in epoxy and hydroxyl groups, C=O (287.7 eV) of the carbonyl group and O–C=O (289.1) in the carboxyl group^30,31^. Figure 5d shows the C1s spectrum of CNGO 5, similar to CNF and GO due to the same composition of matter. However, the intensity of the C1s1 peak attributed to C–C or C–H is relatively improved by incorporating GO^32^. Besides, as shown in the inset of Fig. 5a, the atomic percent result also reveals the increase of carbon atomic percent by incorporating GO from 61.99% in pristine CNF to 67.13% in CNGO composite.

**Figure 6:**
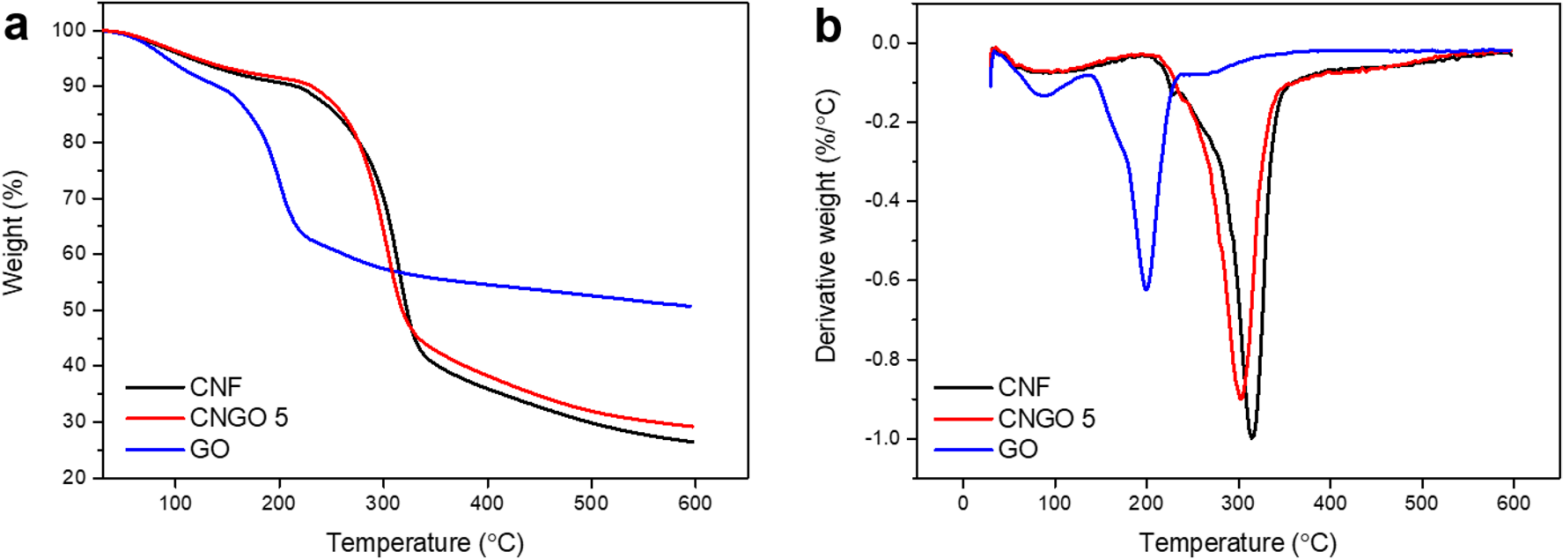
Thermal properties of the pristine CNF, CNGO 5, and GO: (**a**) TGA and (**b**) DTG.

Figure 6 shows TGA and derivative thermogravimetry (DTG) thermograms of the pristine CNF, CNGO 5, and GO. In all cases, the first weight loss occurs due to moisture desorption up to 150 °C. Moreover, as the pristine CNF shows, its onset and peak temperatures of degradation are observed at 212 and 314 °C, respectively. From the CNGO 5, the onset degradation temperature is higher than the pristine CNF at 220 °C due to incorporation between CNF and GO. However, the peak temperature of degradation is lower than 301 °C because of the decomposition of labile oxygen groups of GO, confirmed by the GO. As the TGA and DTG curves show, GO degradation's onset and peak temperatures are observed at 143 and 200 °C, respectively. The low-temperature peak of degradation is caused by the decomposition of unstable oxygen groups such as carboxylic, anhydride, or lactone groups in GO^33,34^. On the other hand, in terms of residue left at 600 °C, the pristine CNF, CNGO 5, and GO have 26, 29, and 51%, respectively. Thus, it seems that the thermal property of GO is transferred to the CNGO hybrid filament.

**Figure 7:**
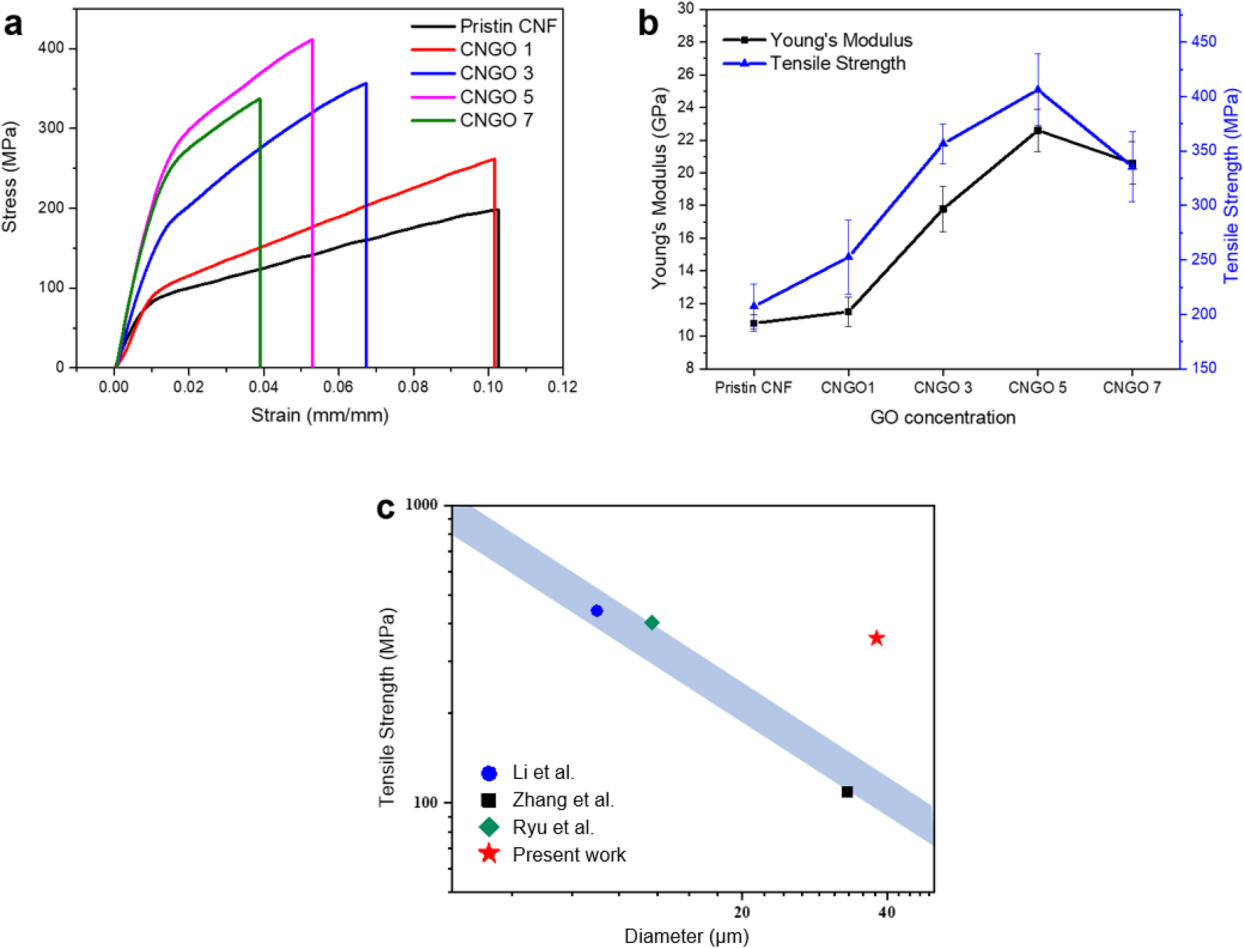
Mechanical properties of CNGO hybrid filaments: (**a**) stress–strain curves, (**b**) variations of Young's modulus and tensile strength with the GO concentration, (**c**) comparison of tensile strength with other reports in terms of filament diameter.

CNGO hybrid filament has different mechanical properties from the pristine CNF with various GO content. The mechanical properties are summarized in Table 1, and Fig. 7 shows the stress–strain curves (a), Young's modulus, and tensile strength changes to the GO concentration (b). Young's modulus and tensile strength were enhanced by increasing the GO concentration up to 5 wt%. The Young's modulus improved from 10.8 ± 0.5 GPa to 22.6 ± 1.3 GPa, and the tensile strength did from 207.5 ± 20.2 MPa to 406.3 ± 33.1 MPa. The yield strength also increased with the GO concentration. However, the elongation-at-break reduced with the GO concentration due to its low fracture toughness^35,36^. The improved mechanical properties might be associated with the well-dispersion of GO in the CNF matrix and plenty of intermolecular hydrogen bonding formed between the GO and CNF, which results in strong interactions between them. On the other hand, at the 7 wt% of GO concentration, the modulus and strength somewhat decreased. The excessive GO over the balanced interaction with CNF could be worked as defects in the blend such that the mechanical properties decreased. The 5 wt% of GO concentration was found as an optimum condition in terms of mechanical properties.

**Table 1:** Tensile test results.

	GO concentration					
	Pristine CNF	CNGO 1	CNGO 3	CNGO 5	CNGO 7	CNGO5 (continuous)
Young’s modulus (GPa)	10.8 ± 0.5	11.5 ± 0.9	17.8 ± 1.4	22.6 ± 1.3	20.6 ± 1.3	22.0 ± 1.4
Tensile strength (MPa)	207.5 ± 20.2	252.7 ± 34.2	356.8 ± 18.3	406.3 ± 33.1	335.6 ± 32	357.4 ± 9.2
Strain-at-break (%)	10.0 ± 1.3	10.5 ± 3.0	7.2 ± 1.4	5.7 ± 0.6	3.8 ± 0.6	5.1 ± 0.4
Yield strength (MPa)	95.0 ± 13.5	103.2 ± 7.2	168.5 ± 6.9	243.3 ± 27.0	259.4 ± 9.9	229.6 ± 8.0
Toughness (MJ m^−3^)	14.3 ± 3.2	17.7 ± 7.2	17.3 ± 4.3	15.2 ± 2.0	8.2 ± 1.0	13.1 ± 1.3

Figure 7c shows the mechanical properties comparison of the manually processed CNGO hybrid filament with other reports. Their Young's modulus and tensile strength values are shown with the filament diameters. As the diameter decreased by stretching, more alignment could happen in the filament such that the mechanical properties increased^37^. However, the small diameter fiber is not favorable for composites and textiles applications. Thus, although the mechanical properties can be increased by decreasing the filament diameter, it should be limited to a specific range. Once the CNGO filament diameter is reduced within 20 μm by stretching, more than 800 MPa tensile strength and 40 GPa of Young's modulus could be achieved.

With the optimal GO concentration, 5 wt%, the continuous process for CNGO hybrid filament was carried out successfully. It is challenging to implement the continuous process while maintaining the superior mechanical properties studied through the manual process. However, it could be solved by incorporating the stretching process for CNF alignment into the continuous process. At first, the first wheel speed was tuned to the wet-spinning speed, and the second and third wheel speeds were differentiated for 10% stretching ratio, which was limited in this experiment. Then, the overall process speed was adjusted to completely dry the CNGO hybrid filament before winding on the third wheel. If the filament is still wet when it reaches the winding (third) wheel, the overall speed should be reduced, limiting the second and third wheels' speed difference for stretching. The winding (third) wheel was located higher and further than the second one such that it can increase the drying time without contacting any rollers, which will help the filament be a round shape (see Fig. 1a).

**Figure 8:**
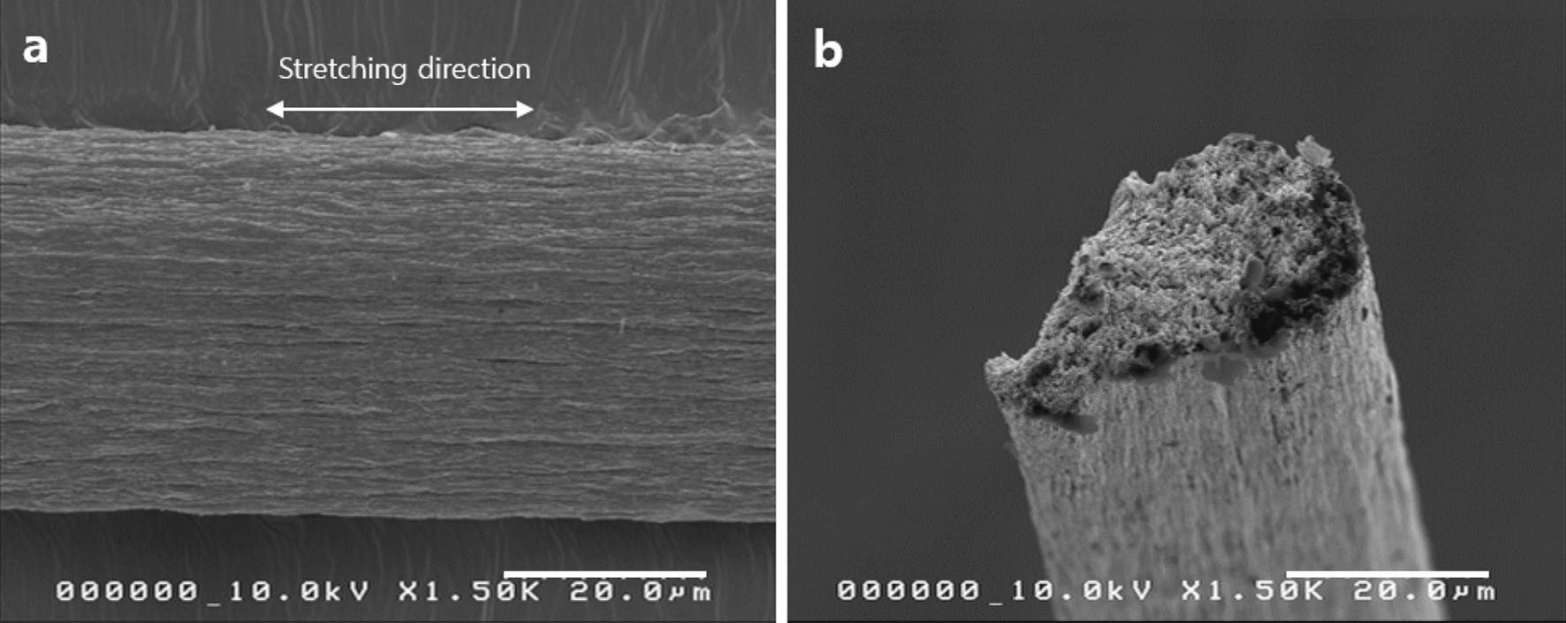
The SEM images of the continuously processed CNGO hybrid filament: (**a**) surface and (**b**) fractured surface.

**Figure 9:**
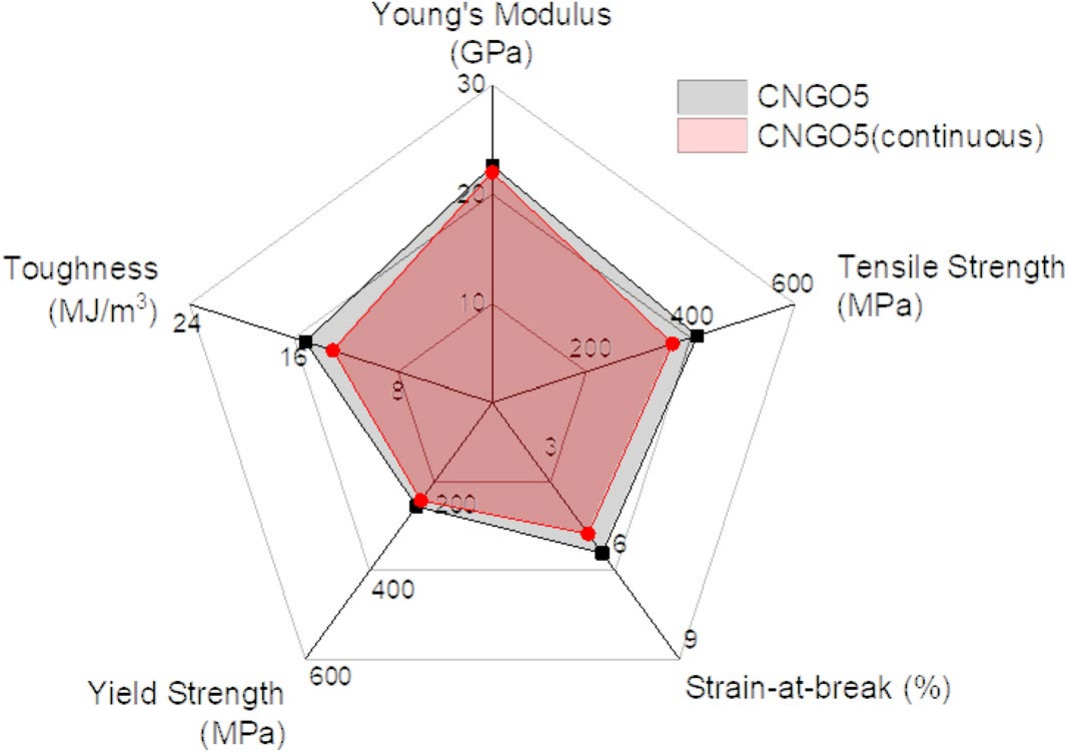
Mechanical properties comparison chart of the CNGO5 hybrid filaments by the manual and continuous processes.

Figure 8 shows SEM cross-sectional and surface images of the CNGO hybrid filament by the continuous process. The cross-section shows that the filament has a circle shape. The surface also shows that CNGO hybrid materials well-arranged in the longitudinal direction by the continuous process. Its mechanical properties are listed in Table 1, and Fig. 9 exhibits the mechanical properties of the filaments prepared by the manual and the continuous processes. The CNGO hybrid filament prepared by the continuous process exhibited Young's modulus, tensile strength, and strain-at-break of 22.0 ± 1.4 GPa, 357.4 ± 9.2 MPa, and 5.1 ± 0.4%, respectively. This result is similar to the manual process, which ensures that the outstanding mechanical properties obtained by optimizing the GO concentration were successfully implemented through the continuous process.

**Figure 10:**
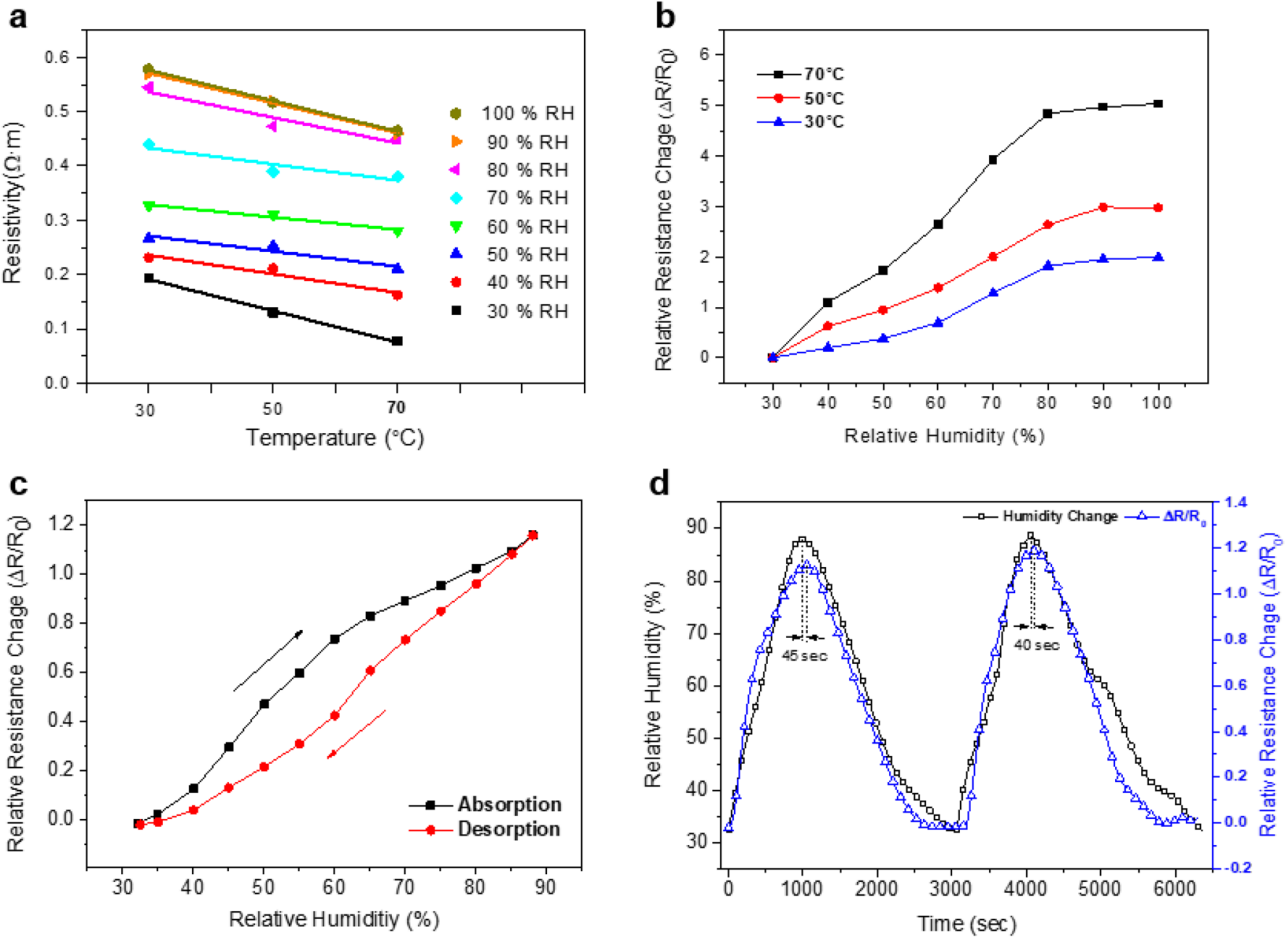
(**a**) Resistivity versus temperature at different RH from 30 to 100%. (**b**) Relative resistance change versus RH with different temperatures. (**c**) Hysteresis characteristics of humidity sensing by CNGO 5 hybrid filament. (**d**) Time response of the sensor with cyclic RH change.

**Figure 11:**
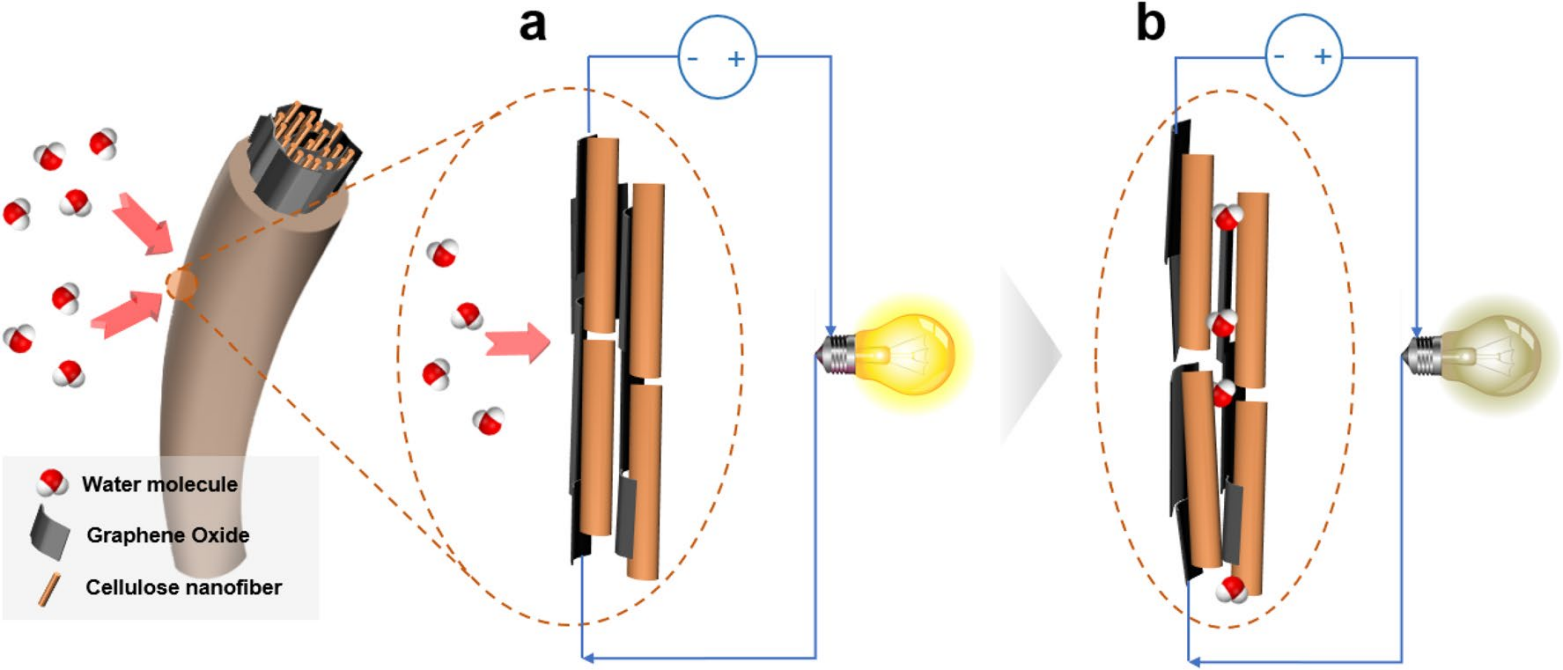
Illustration of the electrical resistance change in the CNGO hybrid filament under humidity change: (**a**) dry condition and (**b**) humidity condition.

The CNGO hybrid filament's electrical resistivity was measured using the four-point probes apparatus for the humidity sensor. The CNGO 5 was selected for the experiment because of its good structural properties. Figure 10a shows resistivity versus temperature curves at different relative humidity (RH) levels from 30 to 100%. 30, 50, and 70 ℃ were chosen for the temperature conditions. The resistivity inherently decreased with the temperature and increased with the RH level. The electrical characteristics of the CNGO hybrid filament are associated with the semiconducting behavior of the filament. CNF is typically hydrophilic because of numerous hydroxyl groups on its surface, and GO a monolayer sheet of graphite with various functional groups with oxygen atoms such as hydroxyl, carboxyl, carbonyl, and epoxide groups. GO can be easily dispersed to form its network in the CNF matrix as significant support of the filament^16,17,38^. Thus, the CNGO filament can possess a semiconducting behavior due to the GO networks in the CNF matrix^39,40^. Electrons can flow through the GO networks in the filament over bandgap energy by given voltage from the four-point probe system. The bandgap energy tends to decrease as the temperature increased. It can be explained via the electrical behavior of the filament characterized by the resistivity measurement with the temperature change under the fixed humidity condition. Furthermore, when the humidity increases, the filament's hydrophilic functional groups attract water molecules into their network structures from the surrounding. Then, the filament can be partially swollen by the water molecules, which leads to cracks on the GO networks to increase the filament resistance (Fig. 11).

Figure 10b shows a clear comparison of the humidity sensing behaviors depending on temperature. For this, a relative resistance change was calculated by the following equation:

2 $$ {\text{Relative}}\;{\text{resistance}}\;{\text{change}} = \frac{{R - R_{0} }}{{R_{0} }} $$

In this study, R_0_ is used as resistance at 30% RH in each temperature environment for the initial condition. It is represented that the relative resistance change increased by the RH increased in all temperature range. Especially in the case of 70 ℃, the relative resistance change from 30 to 100% RH increases by more than four times compared to the 30 ℃. It can be explained based on the equation of relative resistance change. First, the initial resistance (R_0_) of the CNGO hybrid filament showed a lower value as the temperature increased, shown in Fig. 10a. Secondly, the resistance change (R − R_0_) led to a relatively significant value due to the increased kinetic energy of water molecules that cause partially swollen with rising temperature.

Figure 10c shows the humidity sensing hysteresis of the CNGO 5 hybrid filament. The relative resistance change was measured by increasing and decreasing the RH between 30 and 90% at 30 ℃. Again, the absorption curve is ahead of its desorption curve. The hysteresis occurred because the hybrid filament needs time for removing water molecules from the filament surface.

Figure 10d shows the time response of the relative resistance change of the hybrid filament under cyclic RH change. A commercial hygrometer signal was also plotted for comparison. As one can see, the relative resistance change of the hybrid filament nearly follows the RH signal caught from the hydrometer. Note that 45 and 40 s response time delays were observed at the first and second peaks of between two curves. This time delay range is acceptable for humidity sensing. Thus, the CNGO hybrid filament is suitable for humidity sensing with a quick response time to be embedded in composites.

## Conclusions

Wet-spun CNGO hybrid filaments were successfully fabricated by manual and continuous processes. The FTIR analysis identified the presence of the hydrogen bond between CNF and GO. In the TGA and DTG analysis, thermal characteristics inherited into CNGO filament from CNF and GO were verified. The tensile test confirmed that the 5 wt% GO concentration in the filament (CNGO 5) showed the maximum Young's modulus of 22.6 ± 1.3 GPa and the maximum tensile strength of 406.3 ± 33.1 MPa. This achievement is better than other reports in the same filament diameter range.

The continuous process was applied for the CNGO 5 hybrid filament by utilizing the wet-spinning, coagulation, washing, stretching, and drying steps. The filament mechanical properties prepared by the continuous process were similar to the manually processed filament results.

By using the CNGO 5 hybrid filament, its humidity sensing behavior was investigated with various temperature levels. The resistivity inherently decreased with the temperature and increased with the RH level. Hysteresis was observed in the relative resistivity change under cyclic relative humidity change, but the humidity response time was acceptable. The humidity sensing mechanism of the filament is associated with the semiconducting behavior of the filament. Therefore, the CNGO hybrid filament has excellent potential for in-situ humidity monitoring by embedding in smart wearable devices and natural fiber-reinforced polymer composites and environmental sensing devices.

## Supplementary Information

Supplementary Video 1.
